# A Review on the Microbial Synthesis of Cadmium-Based Quantum Dots and Their Applications

**DOI:** 10.3390/molecules31203746

**Published:** 2026-10-19

**Authors:** Xiaoju Li, Jianqun Lin

**Affiliations:** State Key Laboratory of Microbial Technology, Shandong University, Qingdao 266237, China

**Keywords:** biosynthesis, cadmium-based quantum dots, CdS, CdSe, CdTe, biosynthetic mechanisms, bioremediation, hazardous waste treatment, application

## Abstract

Cadmium (Cd)-based quantum dots (QDs), noted for their size-tunable optoelectronic properties and remarkable photoluminescence, have attracted considerable interest and found widespread commercial applications. Preparation methods for Cd-based QDs typically involve harsh, environmentally hazardous chemical and/or physical processes, whereas microbial biosynthesis offers a sustainable, green alternative. This review provides an overview of microbial production of Cd-based QDs (cadmium sulfide (CdS), cadmium selenide (CdSe), and cadmium telluride (CdTe)) using wild-type and genetically engineered bacteria, fungi, and viruses, with particular attention to the underlying biosynthesis mechanisms. Key proteins and peptides implicated in catalyzing or promoting QD biosynthesis include cystathionine γ-lyase, threonine dehydratase, sulfide-quinone reductase, phytochelatin (oligoglutathione peptides), and other metal-binding proteins such as metallothionein. The roles of capping agents (e.g., phosphates, L-cysteine, and glutathione), Cd (II) efflux genes, and extracellular polymeric substances are discussed. The application of this process in hazardous waste bioremediation, whereby Cd- or sulfur-containing waste is converted into valuable Cd-based QDs, is also addressed. Diverse applications are summarized, including QD-sensitized solar cells, fluorescent labeling, photocatalytic degradation of organic pollutants, and CdS-bacterial hybrid systems enabling photosynthetic production of organic compounds. This review contributes to a better understanding of Cd-based QD biosynthesis, although further efforts are needed to control synthesis and address biosafety concerns.

## 1. Introduction

Quantum dots (QDs) are zero-dimensional semiconductor nanoparticles that were the subject of the 2023 Nobel Prize in Chemistry. Among various QD materials, cadmium (Cd)-based QDs constitute a classical model system, having been among the earliest synthesized and most thoroughly explored, and they have established a photoluminescence performance benchmark in the QD field as a whole.

**Figure 1:**
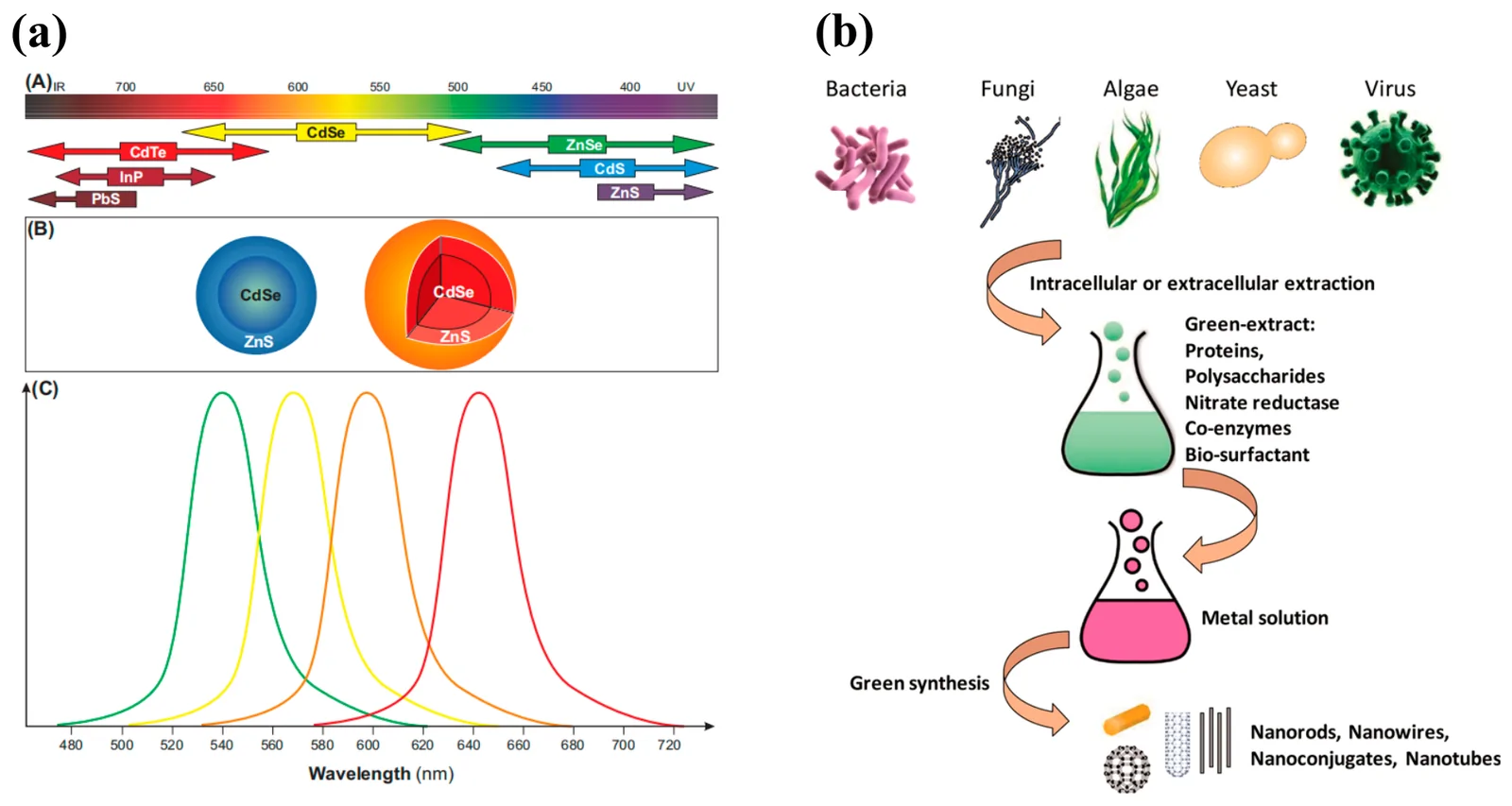
(**a**) Optical spectra of various quantum dots (QDs). The ranges of size-dependent emission wavelengths are as follows: cadmium selenide (CdSe), ~470–660 nm; cadmium telluride (CdTe), ~520–750 nm; indium phosphide (InP), ~620–720 nm; lead sulfide (PbS), ~900 nm; and lead selenide (PbSe), ~1000 nm (**A**). The fluorescence emission spectra of CdSe/zinc sulfide (ZnS) QDs with different particle sizes (3–6 nm in diameter) were obtained using 488 nm excitation (**B**,**C**). Reprinted from Ref. [1] under the Creative Commons license agreement. (**b**) Schematic of the biosynthesis of metal-containing QDs. Adapted from Ref. [12] with permission from the Royal Society of Chemistry (RSC).

The particle size of Cd-based QDs typically ranges from 2 to 10 nm. At this scale, quantum-confinement effects give rise to physicochemical properties markedly different from those of bulk materials. A particularly notable characteristic is the size-dependent tunability of optical emission: luminescence can be precisely modulated from the ultraviolet (UV) to the infrared (IR) region simply by adjusting particle size (representative QDs are shown in Figure 1b). As a general rule, increasing particle size shifts the photoluminescence emission to longer wavelengths (red shift), whereas decreasing particle size shifts it to shorter wavelengths (blue shift). For example, four cadmium selenide (CdSe)/zinc sulfide (ZnS) QD samples with diameters of 3–6 nm exhibit photoluminescence spanning the blue-to-red region, as shown in Figure 1a [1].

Cd-based QDs are conventionally synthesized through colloidal chemical routes in organic media, in which nucleation of semiconductor precursors occurs under high-temperature conditions. However, these organometallic and colloidal approaches involve harsh operating conditions, including high-temperature operation, inert-gas protection, and the use of hazardous organic solvents. Several of these solvents are known carcinogens and may pose explosion risks at the required reaction temperatures [2]. Consequently, the environmental impact of these methods cannot be overlooked.

Given the excellent performance of Cd-based QDs and the toxicity of conventional synthesis methods, green methods using biological systems have been extensively explored [3,4,5]. Biosynthesis and purification of QDs can be achieved using biological resources and nontoxic reagents, without requiring harsh reaction conditions, offering a green, biocompatible strategy for QD fabrication. Furthermore, QD biosynthesis is considered a bottom-up process, involving redox reactions between metal ions (Cd^2+^) and biological molecules (e.g., proteins, sugars, and enzymes) derived from microorganisms. Biosynthesized QDs do not require additional stabilizing agents as the microorganisms or their extracts serve as both stabilizers and capping agents [6,7,8]. Notably, biologically prepared CdS QDs exhibit lower cytotoxicity than chemically synthesized QDs, an effect largely attributed to their surface organic layer (comprising lipids, amino acids, proteins, and nitrate groups), which appears to result in more effective stabilization and capping by proteins [9,10,11].

Diverse organisms, including yeast, algae, fungi, bacteria, and plant extracts, have been explored for QD biosynthesis (Figure 1b) [12,13,14,15,16]. The following sections focus specifically on the biosynthesis of Cd-based QDs using these biological systems.

## 2. Microbial Synthesis of Quantum Dots

### 2.1. CdS Quantum Dots

Dameron et al. first reported that yeast could be used to synthesize cadmium sulfide (CdS) QDs [17]. This discovery spurred extensive research into the use of microorganisms for the synthesis of various types of sulfide QDs. To date, numerous environmental and model microorganisms have been exploited for CdS QD biosynthesis owing to their cost-effectiveness and strong environmental adaptability.

*Escherichia coli*, one of the most commonly used bacterial hosts, has been extensively explored for CdS QD biosynthesis owing to its fast growth under simple cultivation conditions and ease of genetic manipulation. The biosynthesis of CdS QDs has been successfully achieved using recombinant *E. coli.* Foreign genes, such as those encoding γ-glutamylcysteine synthetase, cysteine desulfhydrase [18,19,20], or a CdS-binding peptide [21], were introduced into genetically engineered *E. coli* for CdS QD biosynthesis. Phytochelatin (PC) and metallothionein (MT), both known for their strong heavy-metal-binding ability and widely used for heavy metal removal, have also been introduced into *E. coli* for CdS QD biosynthesis. Engineered *E. coli* overexpressing PC synthase (PCS) and/or MT has enabled rapid intracellular CdS QD production [22,23]. Cd-based QDs can generally be produced intracellularly or extracellularly using *E. coli* through various genetic techniques, with more than 12 h required for their production [24,25].

In addition to *E. coli*, various other biological systems have been explored for CdS QD biosynthesis, including metal- and sulfur-reducing bacteria [26,27,28,29], aerobic bacterial species (*Pseudomonas putida*, *Acidithiobacillus* sp., *Idiomarina* sp., and *polyextremophilic halophiles*) [30,31,32,33,34], and cell-free extracts of *Pseudomonas chlororaphis* CHR05 [35]. However, a relatively long time (even several days) is needed for the biosynthesis of CdS QDs, particularly using certain extremophile microbes [30,31,32,33,34].

**Figure 2:**
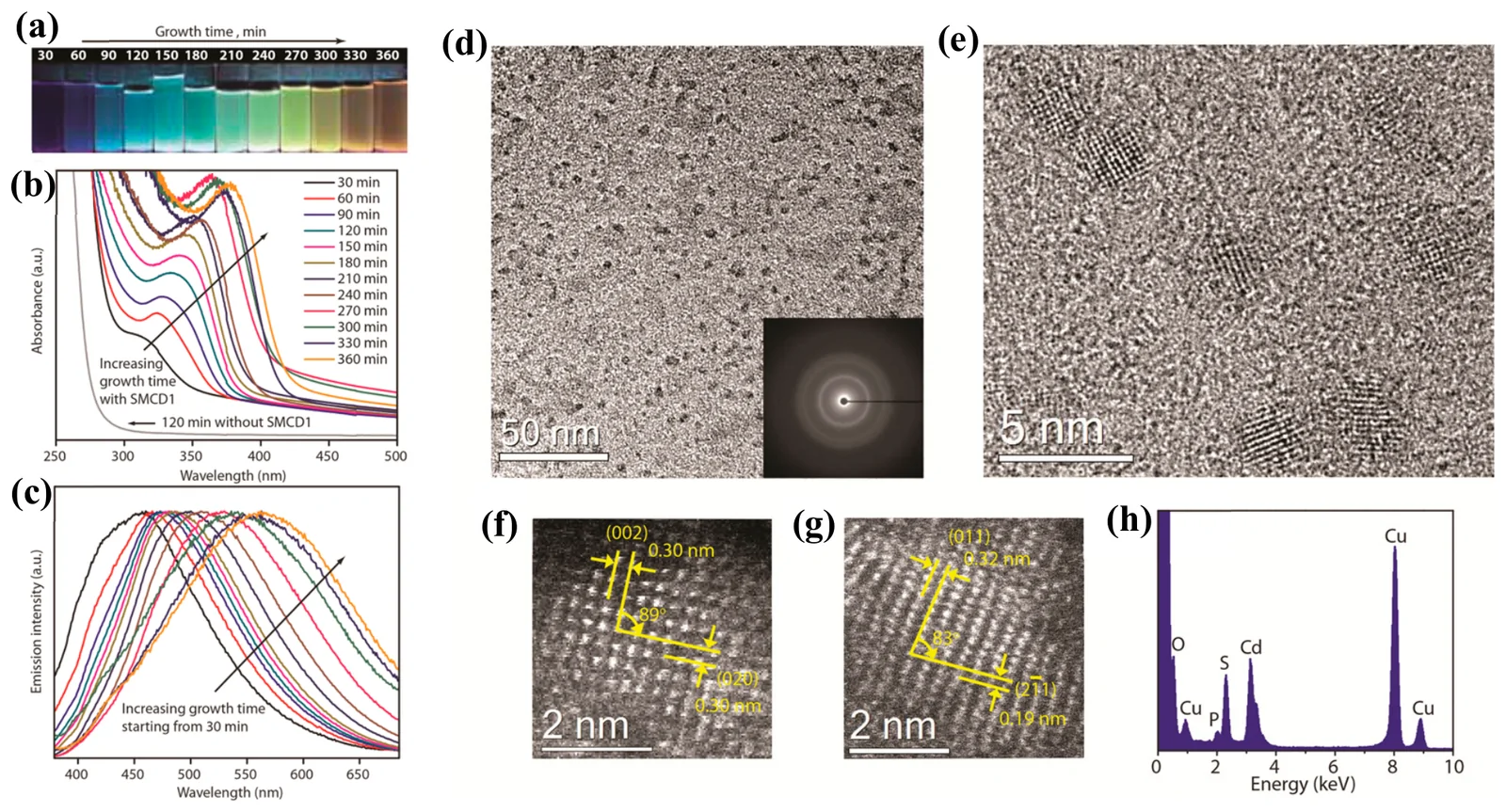
Typical CdS QDs biosynthesized by bacteria (strain *Stenotrophomonas maltophilia*): (**a**) photograph illuminated under UV light; (**b**) UV–vis absorption spectra; (**c**) fluorescence emission spectra (350 nm excitation) of bacterial culture supernatants containing CdS QDs at various growth times. Purified CdS QDs after 60 min of growth were characterized by transmission electron microscopy (TEM): (**d**) bright-field TEM image; (**e**) high-resolution TEM (HRTEM) image; (**f**,**g**) high-angle annular dark-field scanning TEM (HAADF-STEM) images; and (**h**) energy-dispersive X-ray spectroscopy (EDS) analysis of CdS QDs. Adapted from Ref. [36] with permission from the Royal Society of Chemistry (RSC).

Figure 2 shows the typical biosynthesis of CdS QDs using bacteria (strain *Stenotrophomonas maltophilia*), with cadmium acetate [Cd(CH_3_CO_2_)_2_] as the Cd source and L-cysteine as the sulfur source and capping agent [36]. Photoluminescence from CdS QDs was retained in the culture supernatants after removal of the cells via centrifugation. As growth time increased (from 30 min to 360 min), the size of the CdS QDs gradually increased, and the fluorescence gradually shifted from the blue–green region to the yellow–red region (Figure 1a). The size of synthesized CdS QDs ranges from 2 to 5 nm (Figure 2d–g), and they exhibit good dispersion in solution. CdS QDs dispersed in solution exhibit absorption at approximately 350 nm in the UV band (Figure 2b), with substantial fluorescence emitted at 450–550 nm (Figure 2c).

The rapid extracellular biosynthesis of water-soluble CdS QDs via microbial routes is essential for practical industrial applications. To date, several bacterial strains have been reported to biosynthesize CdS QDs extracellularly in less than 3 h. One such strain is *Stenotrophomonas maltophilia* (SMCD1), isolated from mountaintop soil, which exhibits strong resistance to cadmium ions (Cd^2+^; up to 2 mM) [36,37]. Another representative is *Raoultella* sp. X13, isolated from heavy-metal-contaminated soil, which can produce CdS QDs using Cd^2+^ and L-cysteine as substrates [38,39]. *Pseudomonas fragi* GC01 offers a dual-mode capability: it performs intracellular biosynthesis at low temperature (15 °C), whereas at normal temperature (28°C), it switches to extracellular production when Cd^2+^ and L-cysteine are present [40,41]. Finally, *Lysinibacillus boronitolerans* QD4 requires only a few hours to biosynthesize CdS QDs when L-cysteine is added as the sulfur source; after this short incubation period, the characteristic yellow fluorescence of the formed QDs becomes clearly visible [42].

In the microbial biosynthesis of CdS QDs, L-cysteine is frequently used as a sulfur source. Interestingly, in a hybrid system involving the biosynthesis of CdS nanoparticles by *E. coli*, L-cysteine plays an important role in detoxification when the system is exposed to light excitation [43]. More specifically, L-cysteine serves as a substrate for the cystathionine γ-lyase (smCSE) enzyme, leading to H_2_S production. Remarkably, purified smCSE can directly catalyze the synthesis of aqueous CdS QDs from cadmium acetate and L-cysteine, yielding QDs with excellent fluorescence properties [44,45,46].

### 2.2. CdSe Quantum Dots

CdSe QDs are a unique type of QDs that generally exhibit higher photoluminescence quantum yields and better photostability than CdS QDs. The biosynthesis of CdSe QDs has been widely explored using bacteria, yeast, fungi, or earthworms. The synthesis of highly luminescent CdSe QDs by fungi has been reported following incubation of the fungi with a mixture of CdCl_2_ and SeCl_4_ [47]. The intracellular biosynthesis of CdSe QDs has been reported using yeast sequentially cultured with selenite (Na_2_SeO_3_) and Cd [48,49,50,51]. Selenocysteine has been identified as the primary selenium (Se) precursor for CdSe QD biosynthesis [52]. Through genetic techniques, control over CdSe QD yield has been achieved by engineering glutathione (GSH) metabolism in genetically modified yeast cells [53].

**Figure 3:**
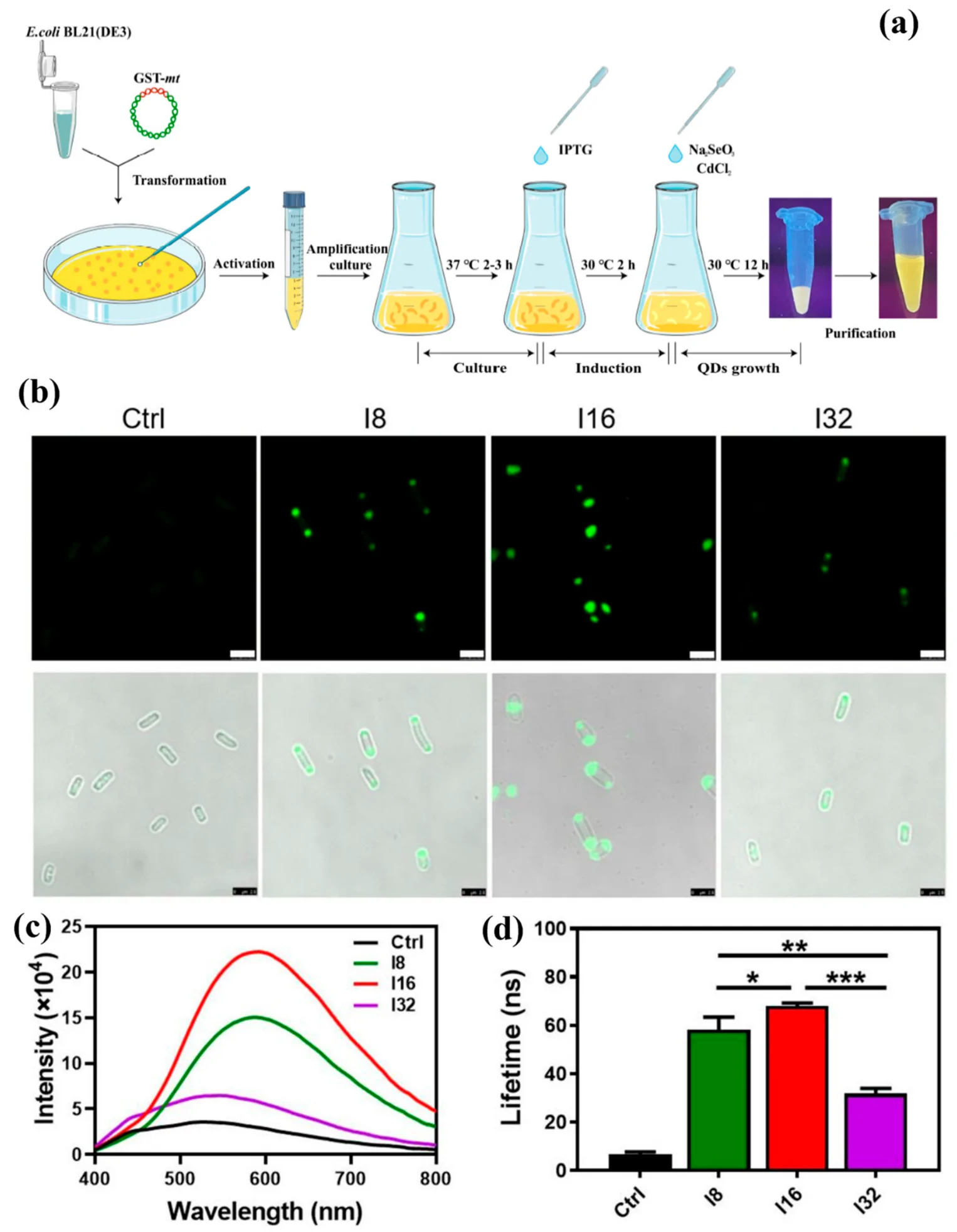
Biosynthesis of cadmium selenide (CdSe) quantum dots (QDs) in metabolically engineered *Escherichia coli* (*E. coli*) with silk protein expression: (**a**) schematic of the preparation of biosynthesized CdSe QDs; inset: purified CdSe QDs emitting distinct yellow fluorescence under ultraviolet light; (**b**) fluorescence microscopy images (excitation: 405 nm; scale bar: 2.5 μm); (**c**) fluorescence emission spectra (excitation: 350 nm); and (**d**) fluorescence lifetimes of biogenic CdSe QDs formed in the *E. coli* cells in which three silk proteins, termed I8, I16 and I32, were individually overexpressed. Data are presented as mean ± s.d. (n = 3). Statistical significance was determined by one-way ANOVA; * *p* < 0.05, ** *p* < 0.01, *** *p* < 0.001. See ref. [55] for details. Adapted from Ref. [55] with permission from Wiley.

*E. coli* has commonly been used for CdSe QD biosynthesis. For example, in vivo biosynthesis of CdSe QDs has been achieved using recombinant *E. coli* expressing MT [54]. Similarly, overexpression of spider silk protein in *E. coli* has enabled the formation of synthetic protein condensates for templated synthesis of CdSe QDs [55]. In addition, CdS_x_Se_1−x_ QDs have been reported to be biosynthesized in *E. coli* using a facile and effective acid-stimulation strategy or by strengthening substrate metabolism to produce more reducing power [56,57]. Typical CdSe QDs biosynthesized using recombinant *E. coli* are shown in Figure 3. The silk protein was overexpressed in bacterial cells and finally formed biomolecular condensates. These condensates can bind metal precursor ions and recruit endogenous reducing agents and other factors, thereby promoting the crystallization and growth of QDs. The purified CdSe QDs exhibit yellow photoluminescence under UV excitation [55].

Reportedly, other microorganisms have also been used to synthesize CdSe QDs. For example, CdSe QDs were successfully biosynthesized using recombinant, photosynthetic *Rhodopseudomonas palustris* expressing MT [58]. In this culture system, after MT expression was induced for 4–6 h, Cd^2+^ (supplied as 3CdSO_4_·8H_2_O, 2 mM) and SeO_3_^2−^ (supplied as Na_2_SeO_3_) were added to produce CdSe QDs. A *gshA*-overexpressing *Shewanella oneidensis* MR-1 strain (*gshA* being related to GSH synthesis) was constructed with the ability to effectively detoxify SeO_3_^2−^ species, resulting in the formation of substantial CdSe QDs throughout the cell [59]. An earthworm has also been reported to biosynthesize CdSe QDs [60].

The GSH metabolic pathway plays an important role in CdSe QD biosynthesis. In a study conducted by Li et al., GSH content was markedly increased in the P*_GAL1_*-*GSH1* cells by induction of the *GSH1* gene, which encodes the enzyme that catalyzes cellular GSH synthesis. Compared with wild-type (WT) cells, P*_GAL1_*-*GSH1* cells exhibited fivefold higher intracellular CdSe QDs fluorescence intensity and a greater ability to biosynthesize CdSe QDs [53]. Zhou et al. found that CdSe QDs were biosynthesized by recombinant *E. coli* expressing MT, indicating that GSH and MT can promote CdSe QD synthesis in microorganisms [54].

### 2.3. CdTe Quantum Dots

**Figure 4:**
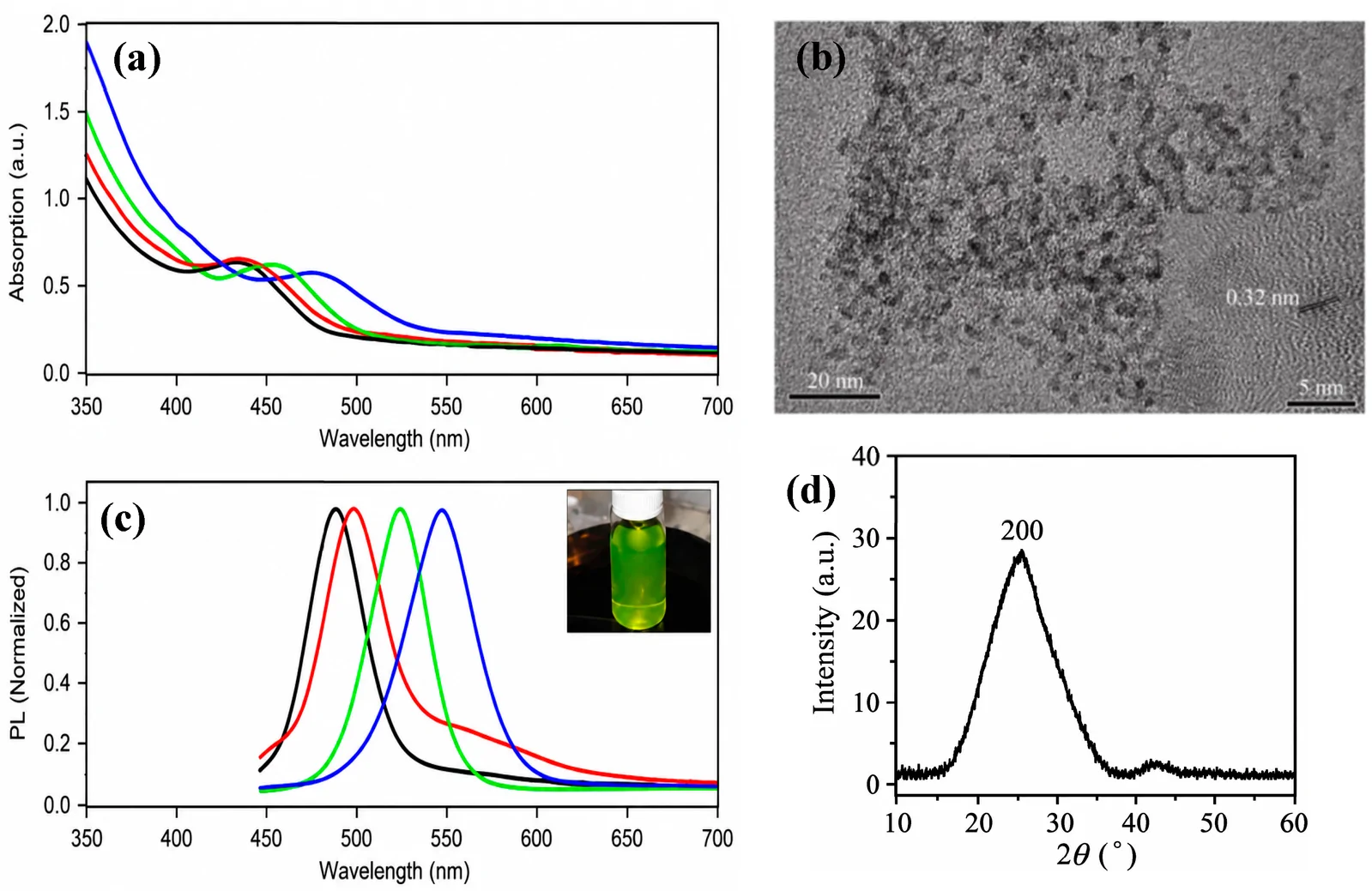
Biosynthesis of CdTe QDs by yeast cells: UV–vis (**a**) and fluorescence (**b**) spectra of the biosynthesized CdTe QDs after 1 (black), 2 (red), 4 (green), and 8 (blue) d. Characterization of the biosynthesized CdTe QDs after 8 d: (**c**) TEM image and (**d**) XRD pattern of the CdTe QDs. Reprinted from Ref. [66] under the Creative Commons license agreement.

Some researchers have also focused on the biosynthesis of cadmium telluride (CdTe) QDs [61,62,63,64,65]. Bao et al. reported the biosynthesis of CdTe QDs using yeast cells [66]. CdCl_2_ (0.04 mol/L), Na_2_TeO_3_ (0.04 mol/L), MSA, and NaBH_4_ were added to the yeast cell culture, which was incubated at 35 °C in a water bath under continuous stirring for several days (2 to 8 d). The resulting CdTe QDs were purified via centrifugation and dialysis and subsequently characterized; the results are shown in Figure 4 [66].

Furthermore, the detoxification system of earthworms has been explored for CdTe QD biosynthesis [67]. *Lumbricus rubellus* earthworms were exposed to soil supplemented with CdCl_2_ and Na_2_TeO_3_ salts. After approximately 11 d of exposure, water-soluble CdTe QDs were extracted from the chloragogenous tissues surrounding the gut of the worm. These QDs exhibited green photoluminescence under UV excitation [67].

### 2.4. Biosynthesis Mechanisms of Cadmium-Based Quantum Dots

**Figure 5:**
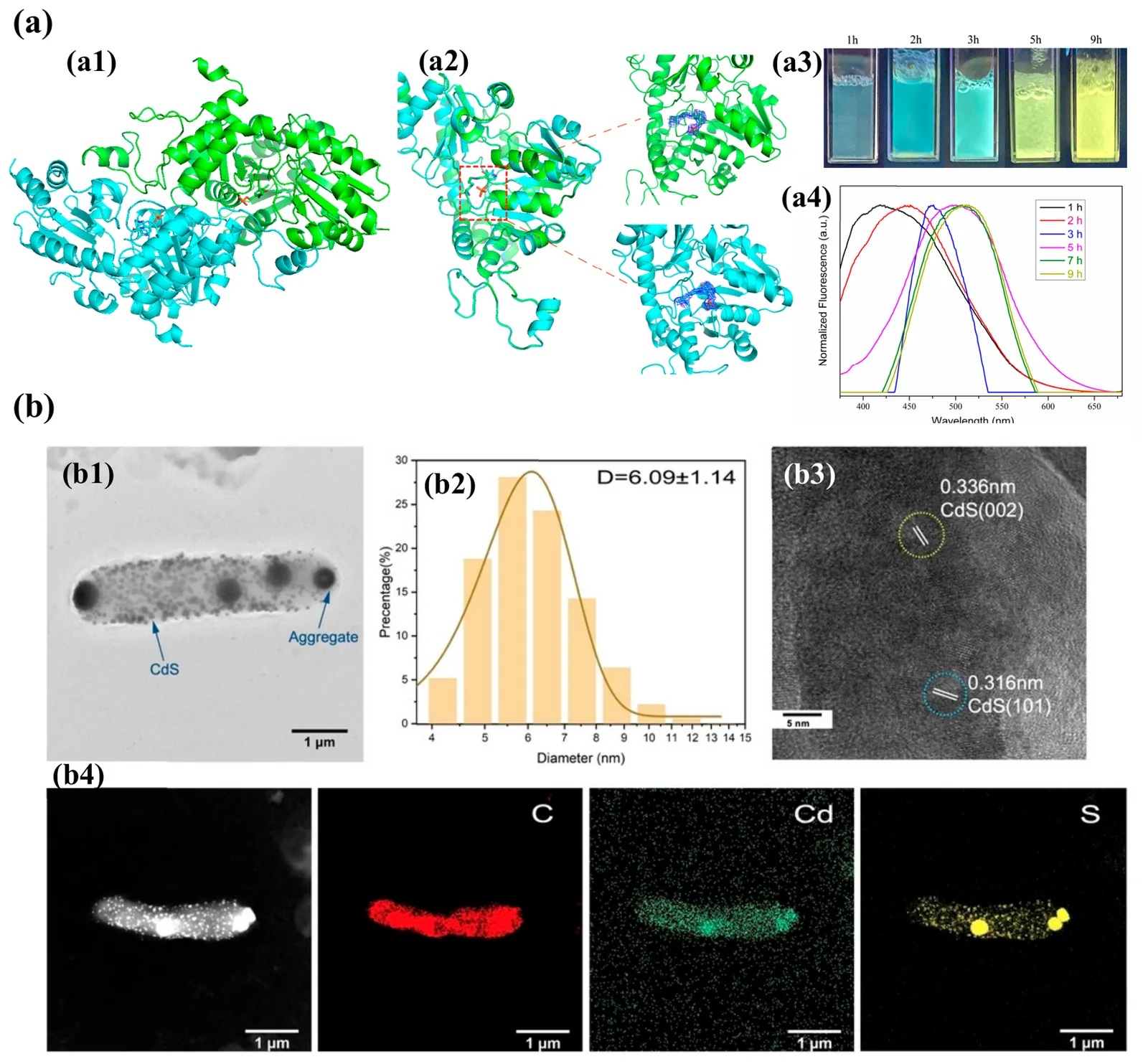
(**a**) The structure of *O*-succinylhomoserine sulfhydrylase (OSH) enzyme, one homologous enzyme of cystathionine γ-lyase (smCSE), a protein that enables the synthesis of cadmium sulfide (CdS) quantum dots (QDs). (**a1**) Apo-structure of the OSH protein, showing a dimer in one asymmetric unit. (**a2**) Molecular interaction map of OSH showing a stable contact with the pyridoxal ring of pyridoxal 5′-phosphate via π–π interactions. (**a3**) Photograph under ultraviolet illumination, and (**a4**) fluorescence spectra of CdS QDs biosynthesized by the OSH enzyme. (**b**) Biosynthesis of CdS QDs by recombinant *Escherichia coli* expressing smCSE: (**b1**) transmission electron microscopy (TEM) image; (**b2**) particle size distribution; (**b3**) high-resolution transmission electron microscopy (HRTEM) image of CdS QDs isolated from bacterial cells; (**b4**) energy-dispersive X-ray spectroscopy (EDS) mapping of CdS QDs attached to the bacterial surface. Adapted from Ref. [46] with permission from Wiley.

The enzyme smCSE (belonging to the cystathionine γ-lyase family) has been identified as a biomolecule that enables the synthesis of CdS QDs. This enzyme uses L-cysteine as a substrate to generate H_2_S, which subsequently reacts with cadmium acetate to form CdS while maintaining nanocrystal dimensions within the quantum-confinement range [44]. To elucidate the mechanism of smCSE-mediated biosynthesis, the atomic-resolution smCSE structure and its homologous protein (O-succinylhomoserine sulfhydrylase, OSH) were analyzed using X-ray crystallography. Pyridoxal 5′-phosphate, commonly known as vitamin B6, is a cofactor employed by the cystathionine γ-lyase family and typically binds to these proteins (Figure 5a). Additionally, dodecapeptide J140 (sequence: SLTPLTTSHLRS) was shown to promote the nucleation and size control of CdS QDs in an aqueous system [68]. Using recombinant technology, a functional domain of J140 was therefore fused to the N-terminal region of smCSE to improve QD synthesis efficiency. Recombinant J140-smCSE protein was expressed and purified using the *E. coli* expression system. Compared with WT smCSE, J140-fused smCSE more effectively accelerated the formation of CdS QDs [45]. Furthermore, Niu et al. achieved precise and selective control over microbial QD biosynthesis by expressing smCSE in engineered *E. coli*. Substantial quantities of CdS QDs were produced in this recombinant *E. coli* when the culture was supplemented with L-cysteine and Cd^2+^ under blue-light activation. Details of CdS QD formation are shown in Figure 5b [46].

Ma et al. found that threonine dehydratase of *P. stutzeri* 273 (psTD) functions as a capping agent during CdS mineralization, controlling the size and structure of CdS QDs. They proposed that threonine dehydratase serves as a critical factor in bacterial Cd resistance and promotes CdS QD biosynthesis by catalyzing the desulfuration of L-cysteine to produce H_2_S. In *P. stutzeri* 273, knockout of the gene encoding psTD resulted in reduced H_2_S generation and decreased Cd resistance, thereby reducing CdS QD biosynthesis [32].

**Figure 6:**
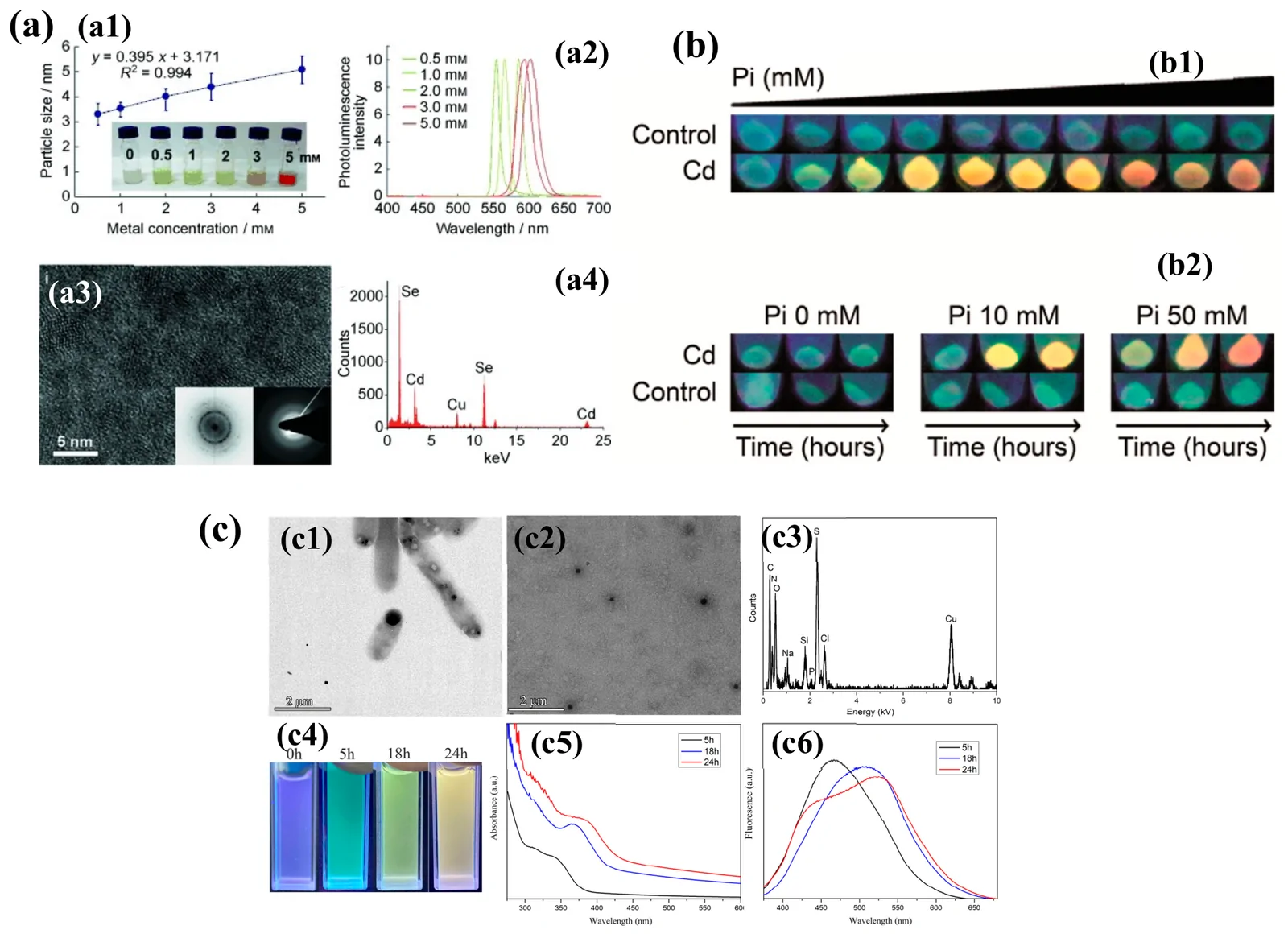
(**a**) Biosynthesis of CdSe QDs by recombinant *E. coli* expressing phytochelatin synthase (PCS) and MT: metal-ion concentration (0.5–5.0 mM) influences the size of the CdSe QDs, giving rise to (**a1**) different colors; (**a2**) different fluorescence emission spectra in the purified samples; (**a3**) an HRTEM image; and (**a4**) an EDS spectrum of purified CdSe QDs. Adapted from Ref. [22] with permission from Wiley. (**b**) Effect of phosphates on CdS QDs biosynthesis—UV-light photographs of *E. coli* pellets: (**b1**) after 24 h of exposure to CdCl_2_ with increasing phosphate concentrations; (**b2**) after exposure to CdCl_2_ and phosphate for different time intervals (2, 8, and 24 h), with controls containing no Cd. Adapted from Ref. [72] with permission from the Royal Society of Chemistry (RSC). (**c**) Sulfide-quinone reductase (SQR)-mediated biosynthesis of CdS QDs: (**c1**,**c2**) TEM images; (**c3**) EDS spectrum of the intermediate sulfur globules; (**c4**) UV-light photographs; (**c5**) absorbance spectra; (**c6**) corresponding fluorescence spectra, all recorded at selected time intervals during CdS QDs formation. Adapted from Ref. [34] with permission from RSC.

PC (an oligoglutathione peptide) and MT (a metal-binding protein) are classic substances with heavy-metal-binding capacity and have been used for heavy-metal removal. Some researchers have explored overexpressing PCS and/or MT in recombinant *E. coli* to promote QD production. QDs with excellent fluorescence performance have been biosynthesized in these recombinant *E. coli.* The fluorescence intensity of CdSe QDs synthesized in cells co-expressing PCS and MT was fourfold stronger than that measured for QDs produced in cells expressing PCS or MT alone (Figure 6a) [22].

Other mechanisms have also been revealed. The intrinsic Cd(II) efflux system in microorganisms affects the biosynthesis of Cd-based QDs. Deletion of the *zntA* gene (Δ*zntA*) in *E. coli* disrupts Cd efflux, resulting in approximately 20% greater intracellular Cd^2+^ accumulation compared with the WT strain. Under treatment with 0.5 mM Na_2_SeO_3_ and 1 mM CdCl_2_ for 4 h, the Δ*zntA* strain yielded 50% more CdS_x_Se_1−x_ QDs relative to the WT strain [69]. Extracellular polymeric substances (EPSs) surrounding bacteria also influence the biosynthesis of Cd-based QDs. CdS QDs biosynthesized by *D. desulfuricans* cells with intact EPS exhibited notably better properties than those produced by cells from which EPS had been removed via NaOH treatment [70,71].

Phosphates have also been reported to promote CdS QD synthesis in biological systems. Venegas et al. evaluated the effect of varied inorganic phosphate concentrations on CdS QD production in WT *E. coli*. Their results showed that at a constant Cd concentration, the fluorescence color of the resulting CdS QDs varied with inorganic phosphate concentration: green at low (1–2.5 mM), yellow at intermediate (5–20 mM), and red at high (40–50 mM) levels (Figure 6b) [72,73]. Furthermore, Li et al. observed substantial extracellular polyphosphates in *Acidithiobacillus* sp., which are often encapsulated by various substances and are characterized as sulfur rich. These polyphosphates have also been confirmed to enhance the biosynthesis of CdS QDs [34].

Li et al. also discovered a new enzyme, sulfide-quinone reductase (SQR), that promotes CdS QD biosynthesis [34]. To biosynthesize CdS QDs, SQR was heterologously overexpressed in recombinant *E. coli* and purified. The purified enzyme was then incubated with NaHS to generate intermediate sulfur globules, followed by the addition of cadmium acetate and GSH. The resulting mixture was placed in a shaker, and CdS QDs with varied photoluminescence properties were obtained at different incubation times (Figure 6c) [34].

**Figure 7:**
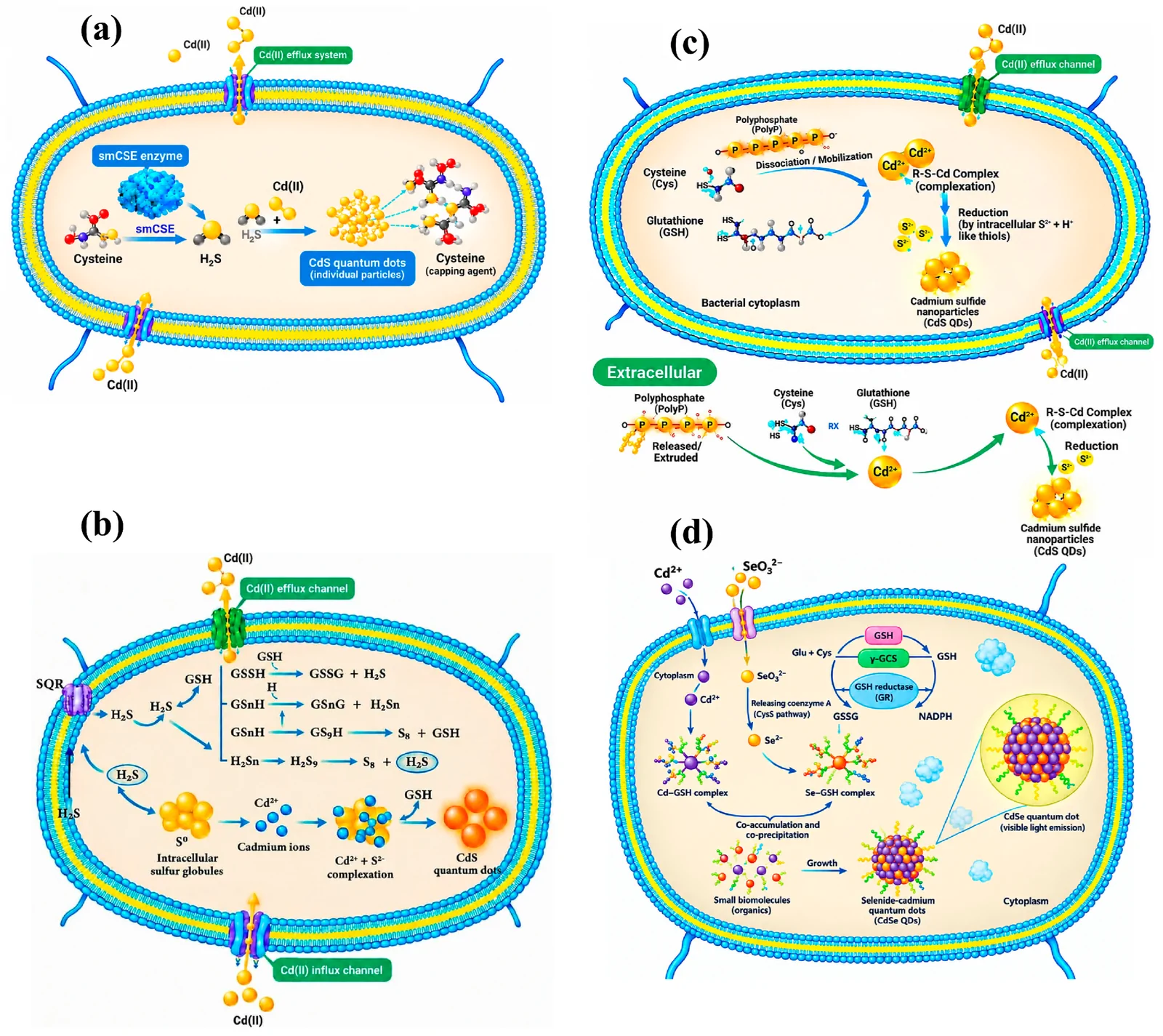
Schematics of (**a**) cystathionine γ-lyase (smCSE enzyme, belonging to the cystathionine γ-lyase family)-mediated biotransformation of cysteine and Cd^2+^ to cadmium sulfide (CdS) quantum dots (QDs); (**b**) sulfide-quinone reductase (SQR enzyme)-mediated biotransformation of glutathione (GSH) and Cd^2+^ to CdS QDs; (**c**) phosphates promote the synthesis of CdS QDs in biological systems; (**d**) GSH metabolism-mediated biotransformation of selenium and Cd^2+^ to cadmium selenide (CdSe) QDs.

Schematics of the biosynthesis mechanisms of Cd-based QDs mediated by certain enzymes or metabolic pathways are shown in Figure 7. Figure 7a shows the smCSE-mediated mechanism, in which cysteine is used as a substrate to generate H_2_S; subsequently, H_2_S reacts with Cd^2+^ to form CdS QDs. Figure 7b shows the SQR-mediated mechanism, in which H_2_S is converted into intermediate sulfur globules that further react with glutathione (GSH) and Cd^2+^ to form CdS QDs. Figure 7c shows that polyphosphate metabolites can promote the biosynthesis of CdS QDs. Figure 7d shows the biotransformation of selenium and Cd^2+^ to CdSe QDs mediated by GSH metabolism in biological systems.

### 2.5. Potential Replacement Quantum Dots

Although Cd-based QDs have remarkable luminescent properties, the toxicity of Cd cannot be overlooked. Biosynthesized Cd-based QDs are often described as green or low-toxicity QDs; however, such claims should be interpreted cautiously. The core toxicological concern is the presence of Cd rather than the synthetic route. Biological capping by proteins, peptides, GSH, or extracellular polymeric substances may reduce Cd^2+^ release under short-term conditions. However, these coatings can degrade under acidic, oxidative, enzymatic, or environmental conditions, potentially leading to Cd^2+^ leakage. Therefore, biosafety should be evaluated using long-term stability tests, leaching assays, ecotoxicological endpoints, and life-cycle assessment rather than assumed from the use of biological systems. Therefore, the synthesis of low-toxicity metal-based QDs with luminescent properties that match those of Cd-based QDs is a major focus of current research. Within the context of microbial biosynthesis, the results reported by Chellamuthu et al. provide a compelling example of an alternative, low-toxicity QDs synthesized through a biosynthetic pathway.

**Figure 8:**
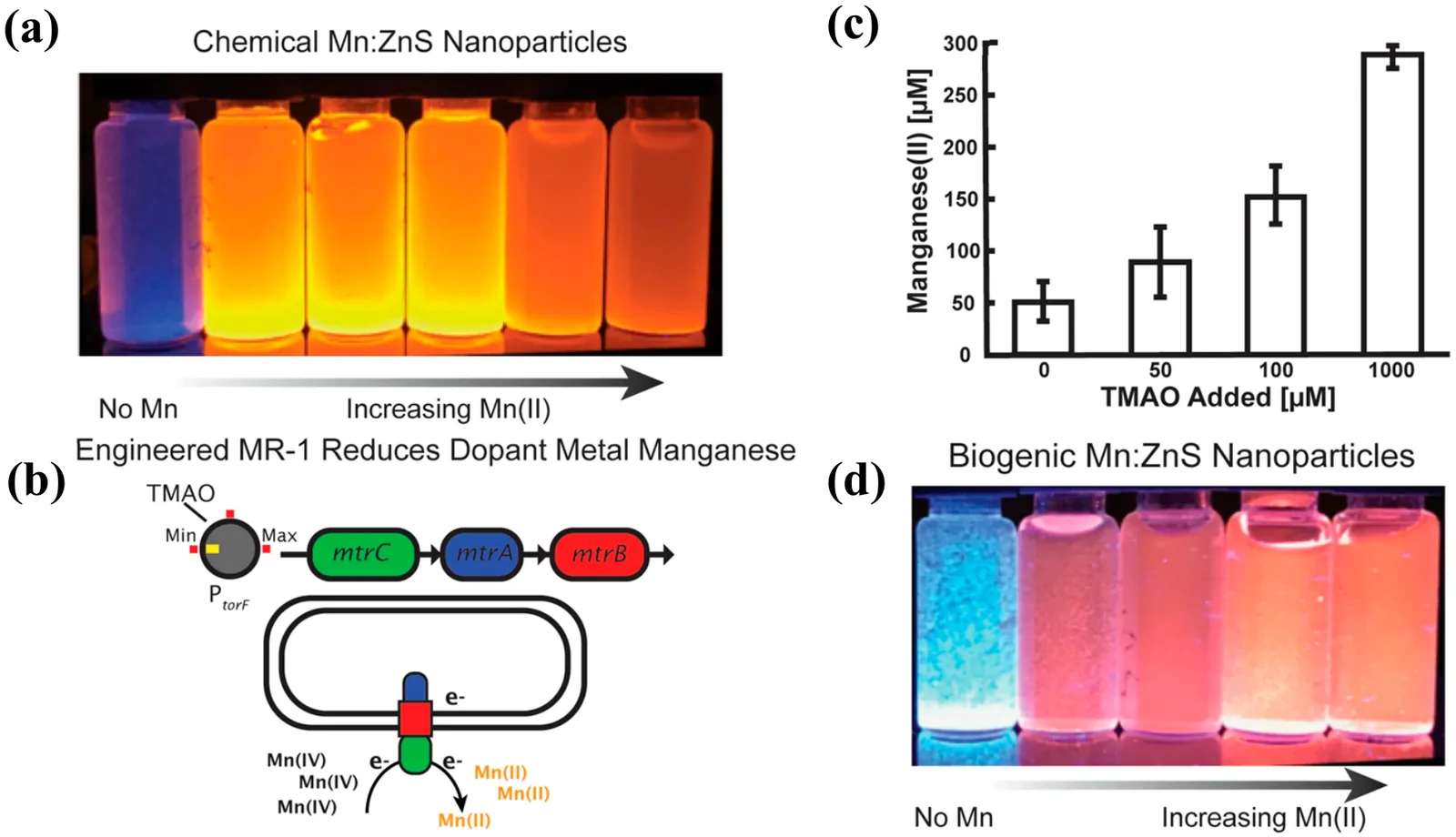
Controlled manganese (Mn) doping of Mn:zinc sulfide (ZnS) quantum dots (QDs) by engineered Shewanella oneidensis: (**a**) Fluorescence of Mn:ZnS QDs produced via chemical synthesis. (**b**) Genetic modification strategy in Shewanella oneidensis for the biosynthesis of Mn:ZnS QDs. (**c**) Mn (II) production in engineered Shewanella cultures (24 h reduction) was linearly proportional to the concentration of the inducer molecule trimethylamine N-oxide (TMAO). (**d**) Mn:ZnS QDs biosynthesized by engineered Shewanella cells show fluorescence similar to that in panel (**a**). Reprinted from Ref. [29] under the Creative Commons license agreement.

Using an engineered strain of *Shewanella oneidensis* MR-1, Chellamuthu et al. modulated bacterial electron transfer for manganese (Mn) reduction to produce Mn-doped ZnS (Mn:ZnS) QDs. The MtrCAB cytochrome complex was expressed under inducer control, enabling precise tuning of the Mn doping level in ZnS QDs. The resulting Mn:ZnS QDs exhibited distinct fluorescence in the visible red region, and their luminescence properties could be altered by varying the Mn doping level, similar to QDs produced by conventional chemical synthesis (Figure 8) [29].

Table 1 presents a comparison of several studies on Cd-based QDs in terms of microorganism/host, QD type, intracellular versus extracellular synthesis, precursor concentrations, synthesis time, particle size, emission wavelength, quantum yield, and purification method.

**Table 1:** List of microbial synthesis of cadmium-based quantum dots.

QD Type	Microorganism	Precursor(s)	Genetic Engineering	Synthesis Time	Size (Å)	Synthesis	Emission Wavelength	Quantum Yield	Purification Method	Ref.
CdS	*Candida glabrata* *Schizosaccharomyces pombe* (yeast)	Cd salts	γ-glutamyl peptides (PCs)	-	~20	Intracellular	~460 nm	-	By ultrafiltration (Amicon PM 30, Merck, Germany) followed by filtration (0.2 μm filter)	[17]
CdS	*Escherichia coli* (bacteria)	CdCl_2_	Phytochelatins (PCs) synthase	6 h –24 h	~358	Intracellular	430–470 nm	-	Cell crushing via ultrasonication and centrifugation for the release of CdS QDs, followed by filtration of the supernatant (0.45 µm filter)	[19]
CdS	*Escherichia coli *(bacteria)	CdCl_2_ cysteine	Cysteine desulfhydrase	6 h –24 h	30 –150	Intracellular–Extracellular	-	-	Ultrasonication and centrifugation for the release of CdS QDs	[20]
CdS	*Escherichia coli *(bacteria)	CdCl_2_ Na_2_S	CdS binding peptide	1.5 h –9 h	~60	Intracellular	445–513 nm	-	Lysis and freezing–thawing of cells, followed by purification with anion-exchange resin (DEAE-Sephadex A-50, Cytiva, Sweden)	[21]
CdS	*Escherichia coli *(bacteria)	CdCl_2_ Na_2_S	Phytochelatins (PCs)	4 h	20 –60	Intracellular	~384 nm	0.007%	Freezing and thawing. The released nanoparticles were separated by anion-exchange chromatography.	[23]
CdS	*Pseudomonas* sp. (bacteria)	CdCl_2_ cysteine	-	3 h –72 h	125 –300	Intracellular–Extracellular	Green to red	-	Using a strong chelating agent (i.e., ethylenediaminetetraacetic acid)	[30] [40]
CdS	*Halobacillus* sp. (bacteria)	CdCl_2_ cysteine	-	24 h –32 h	44 –50	Intracellular–Extracellular	Blue to red	23.53%	Filtration (0.2 μm filter), followed by precipitation with ethanol:water (1:1), centrifugation, dialysis, and concentration using a 3 kDa filter (Millipore, Merck, Darmstadt, Germany)	[33]
CdS	*Acidithiobacillus* sp. (bacteria)	(CH_3_COO)_2_Cd glutathione	-	48 h –72 h	~50	Extracellular	450–550 nm	-	Centrifugation, filtration (0.2 μm filter), and gravity-feed size exclusion chromatography (PD-10)	[34]
CdS	*Stenotrophomonas maltophilia *(bacteria)	(CH_3_COO)_2_Cd cysteine	-	2 h –6 h	27.5 –33.6	Extracellular	450–600 nm	2.08%	Centrifugation, dialysis (3500 MWCO) with ultrapure water, and gravity-feed size exclusion chromatography (PD-10)	[36]
CdS	*Lysinibacillus boronitolerans* (bacteria)	CdCl_2_ cysteine	-	2 h –4 h	~50	Intracellular–Extracellular	450–500 nm	-	-	[42]
CdSe	*Fusarium Oxysporum* (fungus)	CdCl_2_ SeCl_4_	-	96 h	90 –150	Extracellular	~440 nm	-	CdSe nanoparticles were treated with distilled ethanol (2:1 v/v)	[47]
CdSe	*Saccharomyces cerevisiae *(yeast)	CdCl_2_ Na_2_SeO_3_	-	10 h –40 h	26.9–63.4	Intracellular	520–670 nm	-	-	[48]
CdSe	*Saccharomyces cerevisiae* (yeast)	CdCl_2_ Na_2_SeO_3_	-	18h –84 h	28	Intracellular	525–600 nm	-	Cell lysis using a lysis buffer and mixing with zirconium oxide beads, followed by homogenization with a bead mill homogenizer, centrifugation, and filtration using a centrifugal filter unit (Amicon Ultra-15; 100 kDa, Merck, Germany)	[50]
CdSe	*Saccharomyces cerevisiae* (yeast)	CdCl_2_ Na_2_SeO_3_	GGlutathione metabolic pathway	>24 h	-	Intracellular	~575 nm	4.7%	Similar to ref 50	[53]
CdSe	*Escherichia coli* (bacteria)	CdCl_2_ Na_2_SeO_3_	Expressing metallothionein (MT)	12 h	<50	Intracellular	~550 nm	2.91%	Cell lysis using a high-pressure cell crusher, followed by centrifugation, filtration (0.22 μm filter), treatment with protease K., concentration using an Amicon Ultra-15 30 KDa filter, and dialysis using a cutoff of 3.5 kD molecular-weight cutoff	[54]
CdSe CdSe_X_S_1-X_	*Escherichia coli *(bacteria)	CdCl_2_ Na_2_SeO_3_	Expression spider silk protein	4 h	16 –20	Intracellular	~550–600 nm	3.09%	-	[55]
CdSe CdSe_X_S_1-X_	*Escherichia coli *(bacteria)	CdCl_2_ Na_2_SeO_3_	Acid stimulation strategy	3.5 h	31 –35	Intracellular	530–610 nm	7.3%	-	[56]
CdSe	*Rhodopseudomonas palustris* (bacteria)	CdSO_4_ Na_2_SeO_3_	Expressing metallothionein (MT)	12 h	~53.7	Intracellular	460–530 nm	3.85%	Cell crushing via ultrasonication, followed by centrifugation and resuspension in ultrapure water (mixed with 1/3 vol of n-octanol). The precipitated QDs were subsequently washed sequentially with chloroform, ethanol and sterile water	[58]
CdTe	*Antarctic bacteria*	CdCl_2_ K_2_TeO_3_	-	24 h –96 h	-	-	Green to red	-	-	[62]
CdTe	*Saccharomyces cerevisiae* (yeast)	CdCl_2_ Na_2_TeO_3_	-	24 h –192 h	20 –36	Extracellular	490–560 nm	~33%	-	[66]
CdTe	*Lumbricus rubellus *(earthworm)	CdCl_2_ Na_2_TeO_3_	-	264 h	18 –29	-	~520 nm	~8.3%	Dispersal of chloragogen cells in deionized water and centrifugation to obtain a solution containing CdTe QDs	[67]

## 3. Bioremediation of Cadmium or Sulfur Waste

Biosynthesis of Cd-based QDs by microorganisms has shown promising applications in the treatment of Cd- and sulfur-containing hazardous waste. Zhang et al. established a sustainable remediation-synthesis strategy using *S. capricornutum* and *M. aeruginosa* as biological hosts. Fluorescent CdSe QDs were efficiently obtained by co-incubating Cd-immobilized microalgal cells with various Se sources [74].

Bakhshi et al. generated CdS nanocrystals by exposing pure and crude industrial CdSO_4_ solutions to EPS from *Bacillus licheniformis*, which served as biosurfactants. Characterization confirmed that the biosynthesized products were cubic CdS crystallites with sizes of 2–10 nm, regardless of the purity of the Cd source [75].

**Figure 9:**
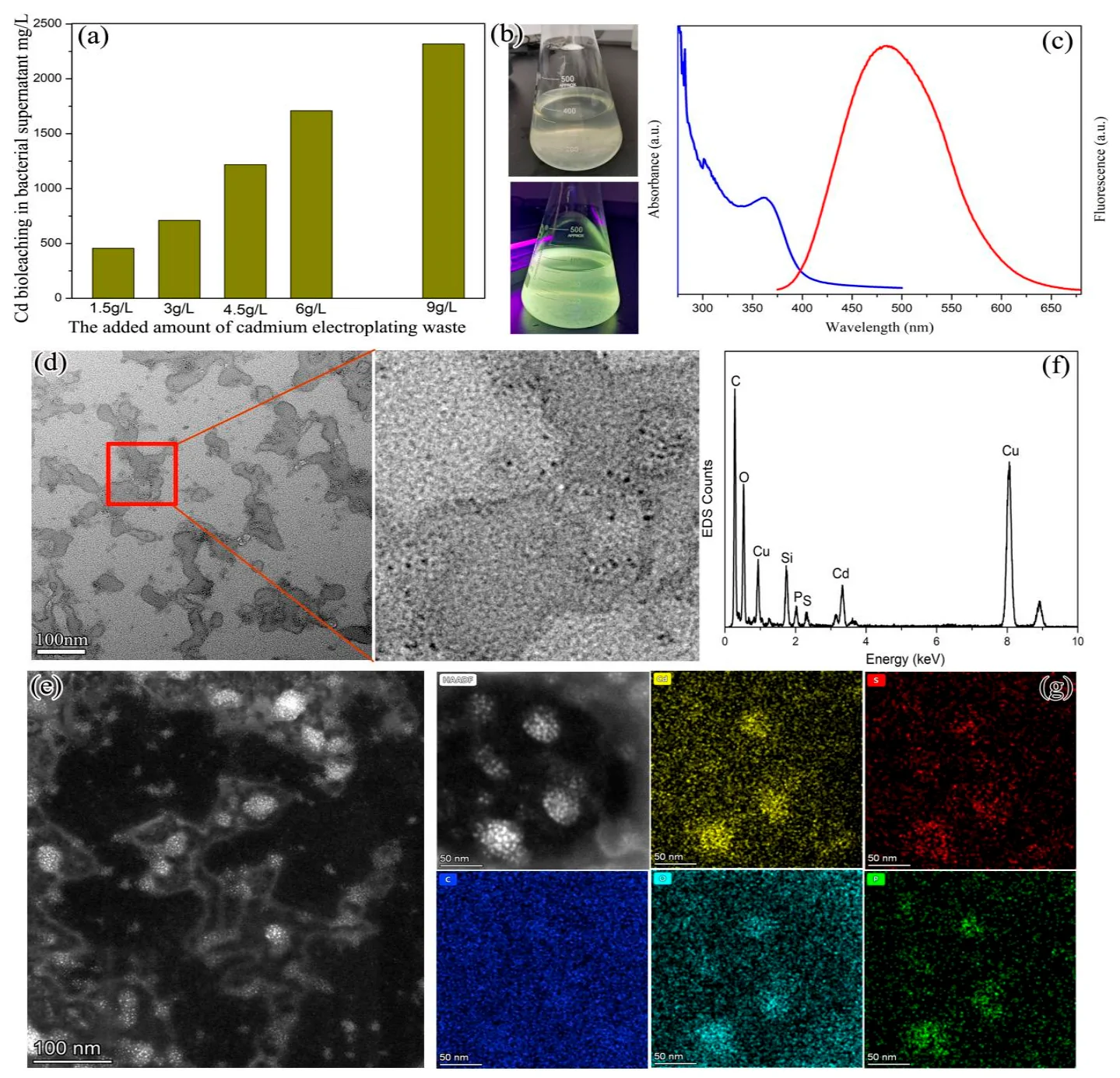
Biosynthesis of CdS QDs from Cd electroplating waste using *Acidithiobacillus* sp.: (**a**) bioleaching of Cd waste into bacterial supernatant as Cd^2+^; (**b**) photograph under room light (**top**) and UV light (**bottom**); and (**c**) absorbance and corresponding fluorescence spectra of CdS QDs biosynthesized from the bioleached Cd^2+^-rich supernatant. (**d**) TEM image; (**e**) high-angle annular dark-field scanning TEM (HAADF-STEM) image; and (**f**,**g**) EDS spectrum or maps of the resulting CdS QDs. Adapted from Ref. [34] with permission from the Royal Society of Chemistry (RSC).

Li et al. reported a straightforward strategy for recycling Cd waste into CdS QDs [34]. This strategy exploited the unique metabolism of *Acidithiobacillus* sp. In particular, Cd electroplating waste was introduced into *Acidithiobacillus* sp. cultures for bioleaching, resulting in Cd^2+^ being bioleached into a bacterial supernatant. The pH of the resulting Cd^2+^-rich supernatant was sequentially adjusted from acidic to neutral conditions via the addition of KOH, followed by supplementation with GSH as a sulfur source. After 3 d of incubation, CdS QDs with distinct yellow fluorescence were harvested (Figure 9) [34].

Furthermore, Sandoval-Cárdenas et al. reported that toxic sulfur-containing waste, generated from the hydrodesulfurization of Mexican sour crude oil, could be used as a sulfur source for the biosynthesis of CdS QDs. Using *Fusarium oxysporum* f. sp. *lycopersici* as the biocatalyst, they converted this sulfur waste into CdS QDs in the presence of 1 mM Cd(NO_3_)_2_ at 30 °C within 24 h. Following paper filtration to remove fungal cells, extracellular QD formation was evidenced by a yellow-colored supernatant, with UV–vis absorption around 300 nm and fluorescence at 510 nm [76].

## 4. Applications of QDs

The potential applications of biosynthesized Cd-based QDs have also been studied in several fields. A few selected examples are highlighted in the following discussion.

**Figure 10:**
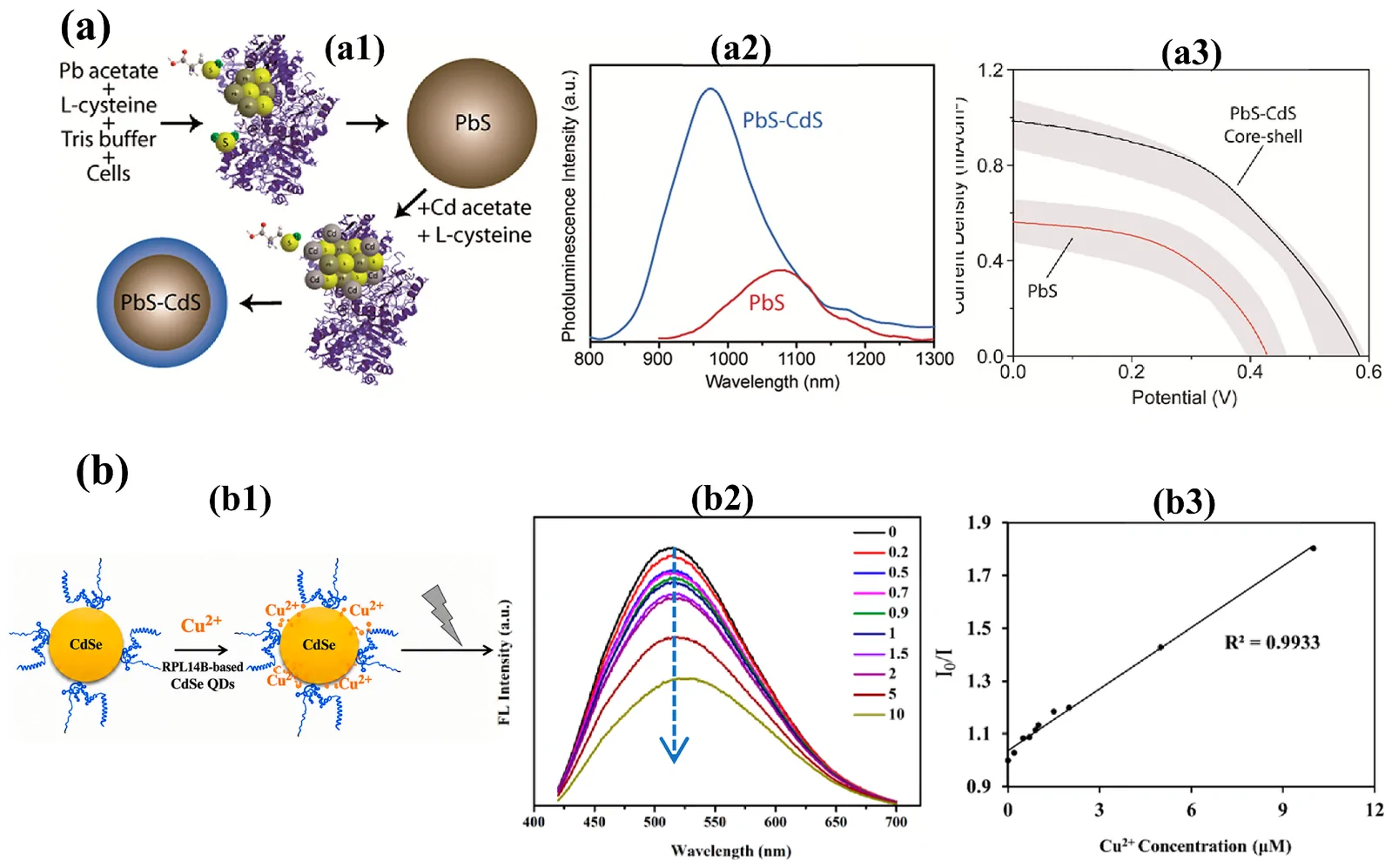
(**a**) Application of biosynthesized PbS/CdS core/shell QDs in quantum dot-sensitized solar cells: (**a1**) schematic of PbS/CdS core/shell QDs fabrication; (**a2**) PL spectra of PbS (red) and PbS/CdS (blue) core/shell QDs; and (**a3**) J-V curves of solar cells with PbS or PbS/CdS core/shell QDs under AM 1.5 illumination. Adapted from Ref. [79] with permission from the Royal Society of Chemistry (RSC). (**b**) Application of biosynthesized CdSe QDs in metal ion (Cu^2+^) detection: (**b1**) fluorescence quenching of RPL14B-based CdSe QDs caused by Cu^2+^; (**b2**,**b3**) correlation between Cu^2+^ concentration and fluorescence intensity of RPL14B-based CdSe QDs. Adapted from Ref. [80] with permission from RSC.

Carrasco et al. attempted to utilize CdS QDs produced by an *Antarctic lithobiontic* bacterium as a photosensitizer material in solar cell applications. The CdS QDs used in the solar cells were approximately 3.0 nm in diameter and consisted of 51.1% Cd and 48.9% sulfide. However, these solar cells exhibited relatively poor performance, with an open-circuit voltage of 162 mV [77]. Órdenes-Aenishanslins et al. obtained CdS/CdSe QDs through the incorporation of L-cysteine, CdCl_2_, and Na_2_SeO_3_ into *E. coli* cultures, enabling precise control over QD synthesis. The biosynthesized CdS/CdSe core/shell QDs, with sizes of approximately 12 nm (CdS) and 17 nm (CdS/CdSe), were used as photosensitizers for QD-sensitized solar cells (QDSSCs). The results indicated that the efficiency of solar cells sensitized with biological CdS/CdSe QDs increased almost 2.5-fold compared with cells sensitized with CdS QDs alone [78]. Spangler et al. achieved biosynthesis of quantum-confined PbS and PbS/CdS core/shell QDs and demonstrated their application in QDSSCs. Using *Stenotrophomonas maltophilia* with lead acetate and L-cysteine, they achieved controlled synthesis of 4 nm PbS nanocrystals. Subsequent exposure to cadmium acetate resulted in biomineralization of a thin CdS shell on the PbS core. The PbS/CdS QDs were fabricated into QDSSCs and yielded an open-circuit voltage of 0.59 V (Figure 10a) [79].

Zhong et al. utilized the yeast ribosomal protein RPL14B, heterologously expressed in *E. coli*, as a biotemplate for CdSe QD synthesis. The resulting CdSe QDs, wrapped in RPL14B, served as turn-off fluorescent sensors for Cu^2+^ detection. As shown in Figure 10b, the fluorescence of these QDs was quenched by Cu^2+^ over a linear detection range of 0.2–10 mM, highlighting their potential for heavy-metal ion analysis [80]. Beyond these applications, CdS QDs biosynthesized using the cyanobacterium *Nostoc carneum* have been used for the photocatalytic degradation of toxic dyes [81], while QDs biosynthesized using *Shewanella oneidensis* and *Bacillus subtilis* have been used as antimicrobial agents [81,82]. Furthermore, Narasimhappa et al. developed fluorescent CdS QDs using *Citrobacter freundii* as a biofactory, enabling sensitive and selective detection of tetracycline via fluorescence quenching [83].

**Figure 11:**
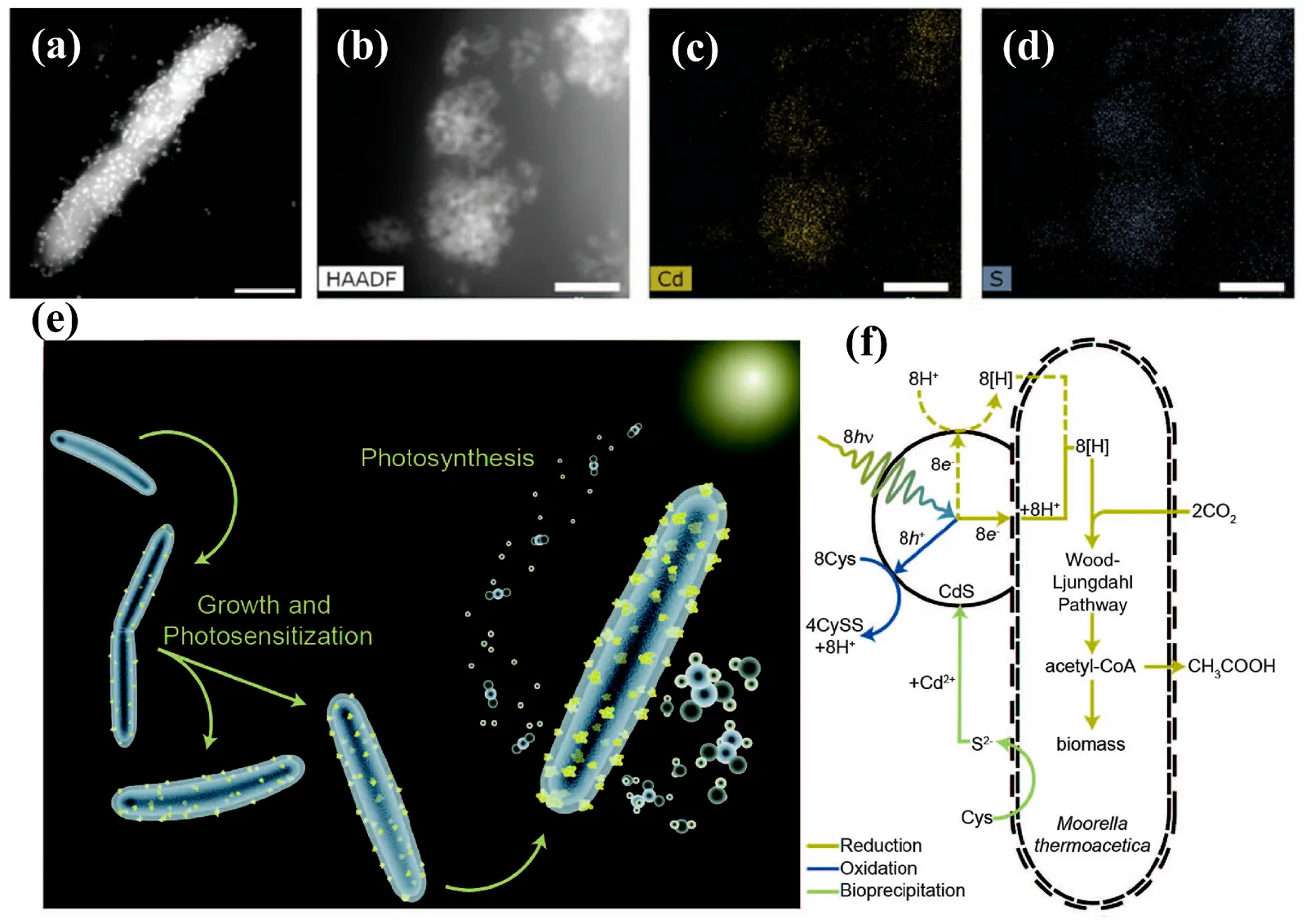
CO_2_-to-acetic acid photoconversion using cadmium sulfide (CdS)–*Moorella thermoacetica *(*M. thermoacetica*) biohybrids: (**a**) High-angle annular dark-field scanning transmission electron microscopy (HAADF-STEM) image of one bacterium with its biologically precipitated CdS nanoparticles, scale bars: 500 nm. (**b**–**d**) HAADF image and energy-dispersive X-ray spectroscopy (EDS) mappings revealed precipitated clusters of smaller nanoparticles (<10 nm) composed of cadmium and sulfur on the cells; scale bars: 50 nm. (**e**) Schematic of the overall system, showing CdS nanoparticles (yellow) biogenesis on the cell surface coupled with light-driven CO_2_ reduction to acetic acid; (**f**) proposed solar-enhanced acetic acid biosynthetic pathway in CdS–*M. thermoacetica* biohybrids. Adapted from Ref. [84] with permission from AAAS.

Sakimoto et al. constructed hybrid systems, namely, CdS–*Moorella thermoacetica* (*M. thermoacetica*) biohybrid. In these systems, CdS induced self-photosensitization of the nonphotosynthetic bacterium, enabling light-driven CO_2_ reduction to acetic acid (Figure 11). The biosynthesized CdS nanoparticles served as light harvesters to sustain cellular metabolism. A two-step pathway was proposed for acetic acid photosynthesis in CdS–*M. thermoacetica* biohybrids: (i) CdS biosynthesis was initiated by the addition of Cd^2+^ and cysteine, and (ii) the bacterium used photogenerated electrons from illuminated CdS nanoparticles to perform photosynthesis [84].

Pi et al. also constructed hybrid systems by biosynthesizing CdS QDs using bacteria and subsequently used them in photosynthetic applications. The biosynthesized CdS nanoparticles produced by engineered *Vibrio natriegens* (*V. natriegens*) were mainly distributed on the cell surface, forming CdS–*V. natriegens* biohybrids. Moreover, these biohybrids could be constructed even using Cd wastewater as the Cd source, while simultaneously achieving a high Cd^2+^ removal efficiency of 95.93%–99.92%. The CdS–*V. natriegens* biohybrids offer a promising route for light-driven chemical synthesis, with potential applications in renewable energy and sustainable bioproduction. Importantly, the resulting biohybrid cells exhibited a higher 2,3-butanediol (BDO) yield compared with pure bacterial cells [85].

## 5. Conclusions

Cd-based QDs, including CdS, CdSe, and CdTe, have become a major focus of nanotechnology research. Conventional synthesis of these QDs is mostly achieved using chemical methods, which employ highly toxic reagents and are environmentally harmful. In contrast, biosynthesis of these QDs uses microbial metabolic pathways under ambient conditions without toxic reagents, offering a greener and more sustainable approach. Diverse biosynthetic strategies, including the use of various microorganisms, functional enzymes or peptides, and genetic engineering techniques, have been exploited for Cd-based QD biosynthesis. These biosynthesized QDs exhibit excellent biocompatibility owing to their good water solubility and naturally modified surfaces bearing macromolecular functional groups.

Microbial synthesis of QDs may play a role in commercial QD production if key limitations, such as low yield and particle size/shape heterogeneity, are addressed. Rapid, controllable biosynthesis of QDs using biological systems, efficient purification and recovery of these biosynthesized QDs, and expansion of their commercial applications are key areas that merit further exploration. For example, although some biosynthetic Cd-based QDs have been applied in solar cells, their broad fluorescence emission bands and relatively low quantum yields compared with chemically synthesized Cd-based QDs markedly hinder their performance in this field. Beyond conventional applications, their use in hazardous waste bioremediation is also a promising research direction, as microorganisms can be utilized to convert hazardous waste into valuable Cd-based QDs. The rational reuse of these transformed QD resources also merits attention in future research. Overall, biosynthesized QDs hold promise as a green and waste-to-value route; however, their practical applications remain at an early stage of development. Addressing the aforementioned challenges, i.e., low yield, poor size and shape uniformity, difficult purification, limited reproducibility, and biosafety concerns, is crucial for advancing this field from proof-of-concept studies to scalable, safe, and sustainable applications.

## Data Availability

No new data were created or analyzed in this study. Data sharing is not applicable to this article.
